# Social network shrinking is explained by active and passive effects but not increasing selectivity with age in wild macaques

**DOI:** 10.1098/rspb.2023.2736

**Published:** 2024-03-13

**Authors:** Baptiste Sadoughi, Roger Mundry, Oliver Schülke, Julia Ostner

**Affiliations:** ^1^ Department of Behavioral Ecology, Georg-August-Universität Göttingen, Johann-Friedrich-Blumenbach Institute for Zoology and Anthropology, Kellnerweg 6, D-37077 Göttingen, Germany; ^2^ Research Group Primate Social Evolution, German Primate Center, Leibniz Institute for Primate Research, Kellnerweg 4, 37077 Göttingen, Germany; ^3^ Leibniz ScienceCampus Primate Cognition, German Primate Center, Leibniz Institute for Primate Research, Kellnerweg 4, 37077 Göttingen, Germany; ^4^ Center for Evolution and Medicine, Arizona State University, Tempe, AZ 85281, USA; ^5^ Cognitive Ethology Laboratory, German Primate Center, Leibniz Institute for Primate Research, Kellnerweg 4, 37077 Göttingen, Germany; ^6^ Department for Primate Cognition, Georg-August-Universität Göttingen, Kellnerweg 4, 37077 Göttingen, Germany

**Keywords:** senescence, social behaviour, social selectivity, primate, longitudinal study, active disengagement

## Abstract

Evidence of social disengagement, network narrowing and social selectivity with advancing age in several non-human animals challenges our understanding of the causes of social ageing. Natural animal populations are needed to test whether social ageing and selectivity occur under natural predation and extrinsic mortality pressures, and longitudinal studies are particularly valuable to disentangle the contribution of within-individual ageing from the demographic processes that shape social ageing at the population level. Data on wild Assamese macaques (*Macaca assamensis*) were collected between 2013 and 2020 at the Phu Khieo Wildlife Sanctuary, Thailand. We investigated the social behaviour of 61 adult females observed for 13 270 h to test several mechanistic hypotheses of social ageing and evaluated the consistency between patterns from mixed-longitudinal and within-individual analyses. With advancing age, females reduced the size of their social network, which could not be explained by an overall increase in the time spent alone, but by an age-related decline in mostly active, but also passive, behaviour, best demonstrated by within-individual analyses. A selective tendency to approach preferred partners was maintained into old age but did not increase. Our results contribute to our understanding of the driver of social ageing in natural animal populations and suggest that social disengagement and selectivity follow independent trajectories during ageing.

## 1. Introduction

The world's human population is ageing rapidly, reaching an estimated 2 billion people over the age of 60 by 2050 [1]. This challenge to societal organizations and healthcare systems can be better met by promoting healthier ageing. One of the keys to healthy ageing may lie in the positive influence of strong social integration on health [2,3]. Concerns therefore arise from research showing that people reduce the size of their social network and their level of social engagement with age [4]. The causes and consequences of this reduction in social engagement are still actively debated [2,4].

Several causal models for reduced social engagement are anchored in the framework of age-related losses (e.g. activity and social exchange theories, reviewed in [4]). Accordingly, individuals experiencing frailty, sickness or loss of social value with age are unable to maintain their social engagement owing to physiological constraints, exclusion by younger generations or diminished self-perception. Although in one case physiological decline translates into social decline while in the other exclusion hampers social engagement, in both cases the reduction in network size and in time spent socially engaged is considered detrimental to wellbeing. However, reduced network size may also result from the tendency of older people to actively focus on preferred partners and positive relationships (e.g. socioemotional selectivity and convoy model theories [5,6]). Recently, evidence is accumulating for decreasing social engagement in non-human animals [7–13] and particularly for social selectivity among non-human primates [14–17].

**Figure 1.**
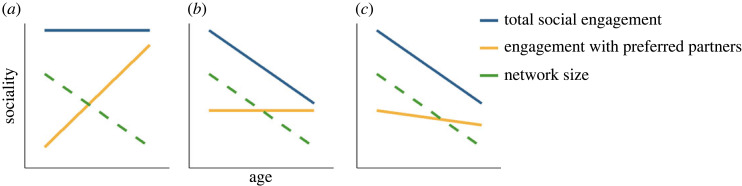
Social selectivity with age can manifest in different ways. (*a*) Social engagement does not change with advancing age, but individuals increasingly prioritize their preferred partners, leading to a reduction in network size. (*b*) Social engagement decreases with advancing age but reduction in network size allows individuals to maintain constant engagement with their preferred partners. (*c*) Social engagement decreases with age, imposing a reduction in network size and engagement with preferred partners, which may still be prioritized relatively to the other potential partners.

The social selectivity in human self-reports can be translated into several predictions for observational studies quantifying animal interaction networks (figure 1). Differences arise from a need to test social features relevant to the study species, and because socioemotional selectivity theory remains vague about how exactly selectivity is expressed at the interaction level. Older individuals may focus their interactions on preferred partners and maintain their overall level of social engagement [15,17], with engagement in preferred partners compensating for the loss of more distant relationships (figure 1*a*). Alternatively, individuals may reduce their overall engagement (figure 1*b*), while maintaining a constant level of engagement with preferred partners [6,13], possibly owing to a loss of interest in less preferred partners. Finally, ageing individuals may decrease their engagement in all relationships, but less so with preferred partners (figure 1*c*) [18]. While increasing selectivity would account for a reduction in network size in the first and second scenarios, the third suggests that another level of constraint is at play.

Although the socioemotional *selectivity* hypothesis focuses on the motivation of the ageing individual in explaining changes in social network and engagement, these changes could also result from several other mechanisms. First, since spatial proximity is a prerequisite for close-range interactions, age-structured spatial distribution may account for age-related social patterns [7]. Individuals that spend relatively more time alone will have fewer opportunities for social engagement, and this may arise from differences in either social or non-social traits (e.g. travelling more slowly, foraging in a different area or spending more time on the ground). Therefore, it is important to test whether *spatial segregation* could drive social interactions on lower scales. Second, individuals may engage in fewer interactions if they experience avoidance or exclusion by others. Crucially, active behaviours typically viewed as actor-driven, such as lower rates of approaching others, may also stem from exclusion. Therefore, actor-centred measures should be complemented with metrics that focus on the partners. Third, older individuals may be unable, or lack the motivation, to socialize, leading to a general disengagement from social interactions across partners in the absence of increasing focus on preferred partners, despite preserved opportunities to do so. Distinguishing between the different hypotheses is a necessary first step in better understanding the drivers and consequences of social ageing. As no behaviour in isolation provides necessary support for one or the other mechanism, investigations should aim to identify the most consistent pattern and a set of minimum necessary conditions.

Age-associated changes in a sample population can result from within-individual ageing or from the selective disappearance of individuals at a younger age based on the feature investigated or other correlated traits [19]. Selective disappearance effects could be strong enough to mask or bias within-individual trajectories, leading to erroneous conclusions about ageing [19]. As sociality often influences health and survival [3,20], less socially integrated individuals could be more likely to be missing from a cohort, particularly among older individuals, creating an apparent association between age and social traits. In the wild, biases due to selective disappearance are of even greater concern, because predation and fatal accidents are more likely to affect individuals in poorer body condition or more peripheral to the group. Captive and food-provisioned populations, which are less likely subject to selective disappearance, have provided compelling evidence for social ageing across [21] and within individuals [17], although patterns may be exacerbated by extreme lifespan and comorbidities (e.g. obesity) that are absent in the wild. Longitudinal data can address this issue by partitioning variance that occurs within individuals versus across individuals [22], and thus provide a more accurate picture of ageing than purely cross-sectional (i.e. one observation per subject over a given age span) or mixed-longitudinal (i.e. consisting of repeated observations over a limited age range for each individual) studies. In this context, longitudinal data on wild animal populations are particularly relevant to determine the occurrence, drivers and consequences of social ageing.

Here, we investigated social ageing in a long-term study population of Assamese macaques (*Macaca assamensis*) living in their natural environment at Phu Khieo Wildlife Sanctuary in Thailand [23]. We focused on females, the philopatric sex in macaques, for which longitudinal social data and more precise age estimates were available. In this population, close female–female affiliative relationships provide benefits such as enhanced tolerance at feeding sites and access to spatially more central positions that may be associated with reduced predation risk [20,24]. In the wild, females must travel daily with their group and experience multiple energetic challenges throughout the year and throughout their lives [25,26], which may exacerbate physiological and physical constraints on sociality compared with captive populations. In this context, two competing predictions can be made. If dynamics in the wild parallel findings from captive and semi-free-ranging populations, we predict that females will reduce their active social engagement in favour of increasing social selectivity with age. On the contrary, if social integration is closely linked to survival, individuals will exhibit no signs of social ageing, and patterns at the population level will suggest an increase in social traits owing to selective disappearance. Using data on female–female interactions collected over 8 years across five social groups, we first aimed to exclude the possibility that (a) spatial segregation would explain age-related changes of closer-range interactions by testing for an increase with age in the time females spent alone. From there on, we tested three hypotheses for social ageing using directed social behaviours to weight the contribution of the partner's and the ageing subject's behaviour. If older females are being excluded, we would expect (b) a decrease with age in the frequency of being approached by others, the number of partners grooming the female, and the time spent receiving grooming. Older females may drive age-related social changes either by becoming increasingly selective or by disengaging from social interactions more generally. To assess these last two hypotheses of increasing social selectivity and social disengagement (c), we would first predict females to actively groom a decreasing number of social partners. In addition, increasing or constant time spent grooming with the remaining partners would be consistent with increasing selectivity (figure 1*a,b*). in the case of decreasing time spent engaging, contrasting the slopes for preferred and all partners will tell whether increased selectivity is nevertheless observed (figure 1*c*). In the absence of evidence for selectivity, the concomitant decrease in the number of partners and the time spent actively interacting with them would suggest social disengagement, which would be further supported by a decrease in the frequency at which females approached others. For all analyses, we report both mixed-longitudinal and within-individual trends, and (d) discuss evidence for inconsistencies.

## 2. Methods

### (a) Study site and study subjects

Data collection was purely observational and non-invasive, without feeding, capture or experimental manipulation, and was authorized by the Department of National Parks, Plants and Wildlife Conservation (DNP) and the National Research Council of Thailand (NRCT) under a benefit-sharing agreement (permit numbers: 0004.3/3618, 0002.3/2647, 0002/17, 0002/2424, 0002/470, 272 0002/4137, 0402/2798, 0402/8707). We studied a population of Assamese macaques living in their natural environment at Phu Khieo Wildlife Sanctuary (PKWS, 16°05′−35′ N, 101°20′−55′ E, >1600 km^2^), part of the more than 6500 km² Western Isaan Forest Complex in northeastern Thailand [25]. These Assamese macaques are seasonal breeders living in hill evergreen forests, with a mating season spanning over the dry season from October to February and births spread from March to August with a peak in mid-May [27].

Each study group was composed of adult females, their immature offspring, and several adult and subadult males, for a total group size between 28 and 98 macaques, with fluctuations resulting from male immi- and emigration, births, deaths and group splits. This study focused on adult females, with females considered adult from the mating season of their first conception (usually at 5.5 years of age) [28]. Year of birth was either known from witnessed birth or inferred by experienced members of the research team based on morphological comparison with individuals of known age. The age distribution had a median (IQR) of 10 (7–16) years and a range of 4–30, consistent with the low survival probability beyond 25 and the maximum documented lifespan in semi-free captive populations of other macaque species [29,30].

### (b) Behavioural data collection

Data were collected on two groups between January 2013 and September 2014, and three groups from January 2015 to July 2019, October 2019 to March 2020 and October 2020 to March 2021 (electronic supplementary material, figure S1). Observations were assigned to the mating or birth season of the corresponding year, creating a season-year time unit (e.g. data collected from October 2019 through February 2020 belonged to the season-year ‘mating 2019′).

During 40 min continuous focal animal protocols [31], we recorded all instances of dyadic grooming interactions between adult females along with the identity of the actor and the receiver. Approaches within 1.5 m of another adult female were also recorded, with the identity of the initiator and receiver. Dyadic agonistic interactions (including aggressions or spontaneous submissions) were recorded during focal animal protocols and ad libitum. Over the study period, 61 adult females were observed for a total of 13 270 h (median (IQR) = 216 (85–332) h per female; electronic supplementary material, figure S2). Females were observed for up to 14 season-years (10 (4–13) season-years per female), for 24 (19–32) h per season-year, and per season-year we observed 39 (33–44) females.

In addition, from January 2015 through March 2021 the identities of group members within 5 m of the focal individual were recorded instantaneously every 10 min during focal animal protocols. A total of 81 969 such instantaneous data points were recorded during 21 722 focal animal protocols (median (IQR) = 5 (2–5) scans per focal follow) from 58 females. For the proximity dataset, females were observed for 10 (6–11) season-years.

### (c) Sociality metrics

#### (i) Grooming out- and instrength, out- and indegree

From the adult female social network, with tie weight reflecting the duration of directed grooming interactions in a group-season-year, we determined two dyad-level metrics reflecting grooming given to and received from a specific adult female partner and four node-level metrics of directed grooming behaviour (out- and instrength, out- and indegree [32]). Strength and degree are direct sociality measures which have been extensively scrutinized for their link to fitness in primates and other mammals [20,33] and pertain to different social strategies [34].

Grooming given to a partner was determined as the time (in seconds (s)) grooming a female partner divided by dyadic observation time (i.e. divided by the sum of observation time for each member of the dyad during which the other member of the dyad was also present and adult). Similarly, grooming received from a partner was determined as the time receiving grooming from that partner divided by dyadic observation time. The resulting dyad-level metrics indicate the strength of directed ties for every pair of females in a group. Individual-level grooming outstrength (given) and instrength (received) were determined as the sum of all female dyadic outgoing and ingoing weighted ties, respectively, allowing females to be compared according to their overall engagement. We determined outdegree and indegree (electronic supplementary material, figure S3) as the number of unique female partners an individual groomed and was groomed by, irrespective of the duration of the interactions.

#### (ii) Rates of selective approach based on tie strength

To test for increasing selectivity with advancing age, we assessed whether females increasingly approached those partners they shared a stronger tie with. We calculated a dyadic sociality index (DSI) for every pair of females in a group based on the time spent in 1.5 m proximity, time spent grooming, the frequency of approach given and the frequency of grooming given [35], irrespective of which partner initiated the interaction. The value for each behaviour in a pair was corrected for dyadic observation time and further divided by the average of all dyadic measures in the group for that behaviour (cf. equation in electronic supplementary material). For each season-year, the index was calculated based on interactions in all previous season-years, so we refer to it as a cumulated DSI. If females are selective, we expected that greater cumulated DSI would predict longer, or more frequent, interactions directed towards the partner in the next season-year, and if females are increasingly selective this association between past and future interactions to strengthen with age.

#### (iii) Rates of approaching and being approached by other adult females

The number of times a focal female approached or was approached by another adult female within 1.5 m was determined per season-year. For one female in one season-year, focal data were collected over only 1 day. This female-season-year was excluded from the analysis. Females directed approximately 1.0 approach per hour (median (IQR) = 20 (11–35) approaches per season-year of roughly 25 h of observations) and received 1.3 approaches per hour (27 (18–44) approaches per season-year).

#### (iv) Proportion time alone

For each instantaneous proximity record, we determined whether the focal female was within 5 m proximity of at least one other adult female. The one female-season-year excluded from the approaches was also excluded here for the same reason. Out of 81 969 data points, 62 842 were recorded with no other female in proximity. With a median (IQR) of 168 (134–230) data points collected per female-season-year, the proportion of records spent alone was 0.77 (0.71–0.83) per female.

### (d) Control variables

To control for social status, dominance Elo scores were determined from winner–loser agonistic interactions [36], and female's Elo score at the end of each season-year or on the last day observed was extracted (see electronic supplementary material, figure S4 for details). To account for possible effects of reproductive state, females giving birth in a given birth season were defined as reproductively active during the preceding mating season and the respective birth season (coded as a binary variable with reference set to not reproductively active). About half of the females were reproductively active per season-year (median (IQR) = 0.56 (0.45–0.67)). Finally, we calculated the number of available partners in each season-year-group as the (number of females in the group – 1, 11 (9–14)).

### (e) Statistical analysis

Response variable and model structures are detailed below. Full model formulae and sample sizes can be found in the electronic supplementary material. Shortened model results for the effect of within-individual age are presented in the main text, while detailed results and mixed-longitudinal model results are provided in electronic supplementary material, tables S1–S10.

#### (i) Grooming outstrength and instrength

The influence of age on outstrength and instrength in the grooming network was modelled with Gaussian error distribution. To improve homogeneity of the distribution of residuals, we applied a square root transformation on the response variables.

#### (ii) Grooming outdegree and indegree

The number of partners an individual was observed grooming and being groomed by, i.e. degree [32], is an unweighted network metric, which is not adequately modelled with a Poisson or normal error distribution because it is biased by sampling effort and reaches an upper limit imposed by group size. To overcome these limitations, we modelled degree as a binomial probability to observe a directed tie from any actor towards any receiver over age, with binomial error distribution including an offset for the log of dyadic observation time (see also [37] using the same modelling approach).

#### (iii) Rates of selective approach based on tie strength

We first aimed to investigate selectivity by testing whether cumulated DSI would be associated with how long females groomed each partner in the next season. However, as the number of partners and time spent giving grooming sharply decreased with age (cf. Results), data lacked resolution. Therefore, we used the number of approaches given to each partner, a more frequent behaviour even at old ages, as the response. Approach directed towards a given partner was modelled with a negative binomial error distribution including an offset for dyadic observation time in the season-year.

#### (iv) Rates of approaching and being approached by other adult females

The tendency of females to engage in social interactions, irrespective of partner identity, was modelled as the count of approaches towards and by others, respectively, with a negative binomial error distribution including an offset for the log of female's observation time.

#### (v) Proportion time alone

The influence of age on the proportion of instantaneous data points an individual was observed alone was modelled with a beta error distribution weighted for the number of records during a given female-season-year. To allow convergence, group was included as a fixed and not a random effect.

#### (vi) General statistical considerations

All analyses were conducted with generalized linear mixed models in R [38] version 4.0.4, with RStudio [39] version 2022.07.02 + 576 [40]. All models included the covariate female age as the key predictor and we furthermore included fixed effects of Elo score as well as reproductive status and season and their two-way interactions, differentiating females that did or did not conceive, did or did not give birth in each mating or birth season, respectively, to control for their effects. The 'number of females in the group – 1' was also included as a fixed effect to account for variation in the availability of partners across season-years. The interaction of actor's age and cumulated DSI was included in the model assessing selective approach. To avoid pseudo-replication, models of grooming outstrength, grooming instrength, approaching others, being approached by others, and time alone included female identity, group and season-year as random intercept effects. Models on grooming outdegree, grooming indegree, and selective approach included the identity of the actor, the identity of the receiver, the identity of the dyad, group and season-year as random intercept effects. Covariates' transformation and model random slope structures are detailed in the electronic supplementary material. Nonlinear effects of age, which we tested *post hoc* upon suggestions by reviewers, did not provide better fit to the data (see electronic supplementary material for details, and figure S5).

### (f) Assessing result robustness to uncertainty about age

Studying wild animal populations implies a degree of uncertainty regarding several individual attributes. As age is central to the present study, we evaluated whether results were robust to possible errors regarding the date of birth assigned to individuals by assigning to each female a symmetric range of possible years around our best estimated year of birth (see electronic supplementary material for details). Results showed that limited uncertainty regarding females’ year of birth would not compromise the conclusions of the present study (electronic supplementary material, figure S6).

### (g) Investigating selective disappearance and its possible influence on the relationship between age and sociality

All individuals are subject to age-related changes in physiology, cognition and possibly social behaviour. Within-individual ageing represents changes in the response as an individual grows older, whereas between-individual ageing represents the association between age and the response at the level of the sample population under study. In a situation where individuals' death is not random but statistically associated to the response variable (i.e. selective disappearance, with or without causality), a between-individual effect is measured even without within-individual ageing. For example, more social individuals may be better buffered against external or intrinsic sources of mortality (e.g. predation), resulting in the ‘selective disappearance’ of less social individuals at an earlier age. Within-individual centring is advocated to remove this confound, because it separates the within- and between-individual effect of age into two independent variables [22,41,42] (see electronic supplementary material for details). Eleven females died during the study (median (IQR) = 21 (15–27) years old).

To investigate whether selective disappearance influenced the relationship between age and sociality, we reran all models after applying within-individual centring. To identify the slopes of within- and between-individual ageing, a first set of models included within-individual-centred ages (representing within-individual changes) and average ages (i.e. an individual mean age over the observation window, representing the between-individual effects). A second set of models included the raw age and the average age to assess the significance of the difference between the within- and between-individual slopes, indicated by the *p*-value of the average age term in these second models ([22], see electronic supplementary material for details).

## 3. Results

### (a) Spatial segregation as a possible driver of changes in social interactions with advancing age

**Figure 2.**
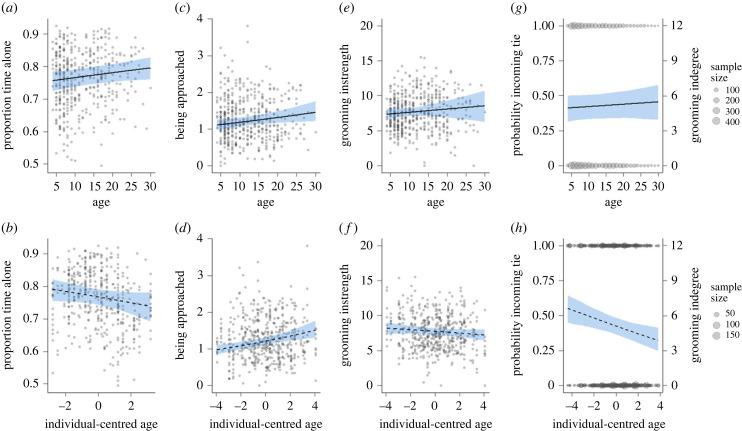
(*a*) Time spent alone slightly increased with age in the mixed-longitudinal analysis (*b*) and did not change within-individual. The possibility that older individuals were being excluded was not supported by (*c,d*) the increasing frequency of being approached, and (*e,f*) the absence of change in grooming received. In mixed-longitudinal analysis, (*g*) the number of partners grooming the subject (indegree) remained relatively unchanged, which contrasted with (*h*) a sharp decrease within-individual. Degree is modelled as the probability of observing a directed tie between a female and every other female in her social group (left axis). To facilitate interpretation, this probability is expressed on the right axis as a count of partners for a group size of 12 adult females with equal observation time. Age is expressed in years in the upper row and frequency is given per hour.

Females were not increasingly spatially segregated with age. Regardless of age, females were typically observed without another adult female within 5 m of them. The proportion of time spent alone showed a marginal 3% increase in mixed-longitudinal analyses. This was not consistent within-individual, which we express for a female on average 15 years old and ageing from 11 to 19 over the 8-year study period. The predicted proportion of time alone showed a small 8% decrease within-individual, associated with large uncertainty (table 1, figure 2*a,b* and electronic supplementary material, table S1). This suggests that with advancing age, females did not spend more time alone, spatially segregated without any partner to interact with. This condition being fulfilled, we next investigated whether passive and active social behaviours changed with age.

**Table 1.** Estimated slopes for the effect of increasing age within and between individuals on the social behaviour investigated (see electronic supplementary material, tables S2–S10 for full model results). Hypotheses tested are increasing spatial segregation, exclusion by partners, increasing social selectivity and disengagement. The significance of the difference between within- and between-individual age slopes is indicated by the *p*-value of the estimate of average individual age in a model including both raw and average individual age terms and suggests selective disappearance.

hypothesis	response	within-individual effect				between-individual effect				slope difference	
		*β*_within age_ ±s.e.	CI_lower_	CI_upper_	*p*	*β*_average age_ ±s.e.	CI_lower_	CI_upper_	*p*	*p*_(*β*-average − *β*-within)_	supported
spatial segregation	proportion time alone	−0.291 ± 0.187	−0.623	0.049	0.127	0.083 ± 0.028	0.023	0.140	0.005	0.072	N
exclusion	being approached	0.317 ± 0.092	0.163	0.495	0.003	0.030 ± 0.027	−0.019	0.083	0.261	0.013	N
exclusion	grooming instrength	−0.720 ± 0.453	−1.623	0.193	0.661	0.536 ± 0.152	0.234	0.861	0.007	0.253	N
exclusion	grooming indegree	−0.661 ± 0.232	−1.067	−0.217	0.008	0.144 ± 0.060	0.025	0.256	0.006	0.004	Y
disengagement/selectivity	grooming outdegree	−0.937 ± 0.204	−1.349	−0.516	<0.001	−0.311 ± 0.050	−0.407	−0.210	<0.001	0.007	Y
disengagement/selectivity	grooming outstrength	−2.564 ± 0.509	−3.590	−1.582	<0.001	−1.162 ± 0.197	−1.527	−0.803	<0.001	0.026	Y
increased selectivity	selective approach	−0.130 ± 0.147	−0.427	0.172	0.377	−0.085 ± 0.037	−0.163	−0.019	0.023	0.786	(N)
disengagement	approaching others	−0.414 ± 0.139	−0.647	−0.158	0.004	−0.082 ± 0.036	−0.148	−0.019	0.037	0.036	Y

### (b) Contribution of social exclusion to patterns of social ageing

Females had fewer partners grooming them, although there was overall limited evidence that they were being socially excluded. The mixed-longitudinal and within-individual analyses revealed that age was associated with a higher tendency to be approached, with a predicted increase of 54% approaches received per hour from 11 to 19 years (table 1, figure 2*c,d* and electronic supplementary material, table S1). Age did not affect the time females spent receiving grooming from female partners (instrength, figure 2*e*,*f*). Finally, the number of partners grooming the subject (indegree) appeared to be relatively constant in the mixed-longitudinal analysis, with females being groomed by an average of six partners. However, this result was not representative of the clear 43% decrease in indegree within individuals (figure 2*g*,*h*). To place this in a socially relevant context, figure 2*g,h* depicts this probability also as a count of partners (i.e. degree) by multiplying age-specific probabilities for each tie by 12, which is the median number of adult females present in a group in a season-year in this study. Therefore, at least one aspect of passive sociality, here the number of partners grooming the subject, decreased with advancing age, whereas the patterns across all three passive behaviours investigated provided only mixed evidence for the social exclusion of ageing females.

### (c) Testing increasing selectivity and increasing disengagement with advancing age

**Figure 3.**
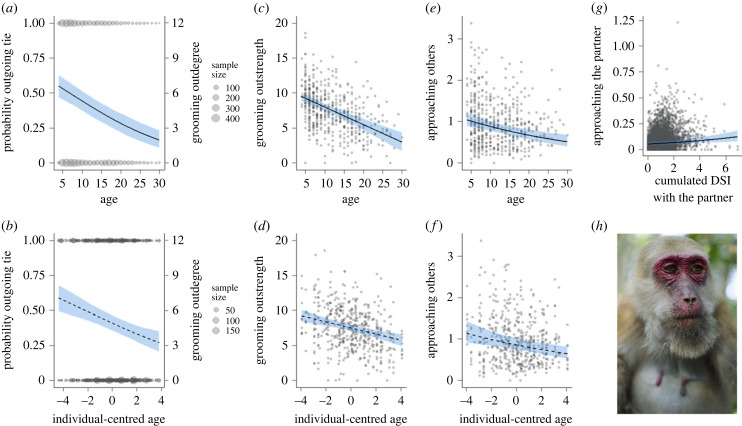
Age was associated with (*a,b*) a reduction in the number of partners females groomed (outdegree). Degree is modelled as the probability of observing a directed tie between a female and every other female in her social group (left axis). To facilitate interpretation, this probability is expressed on the right axis as a count of partners for a group size of 12 adult females with equal observation time. (*c,d*) Grooming outstrength, i.e. total grooming given across all partners, decreased with age. (*e,f*) The frequency with which females approached others decreased with age. (*g*) The frequency of approaches directed at specific partners increased with increasing cumulated dyadic sociality index estimated based on past interactions. (*h*) Nineteen-year-old female Assamese macaque from the study population. Age is expressed in years in the upper row and frequencies are given per hour. DSI, dyadic sociality index.

Although females reduced their number of and time with grooming partners with age, they did not enhance their focus on preferred partners. Instead, they approached partners with whom they shared strong bonds similarly at all ages. To disentangle scenarios of social selectivity, it is necessary to consider the number of partners a female is actively interacting with, the overall engagement across all partners, and interactions directed to preferred partners (figure 1*a–c*). Older females were less likely to groom other females, leading to a marked 61% decrease in the probability of observing a specific outgoing tie for a female ageing from 11 to 19 years of age (figure 3*a*,*b*, table 1 and electronic supplementary material, table S1). Grooming outstrength, a measure of total engagement in grooming given, was also negatively correlated with age, showing a reduction by 48% (figure 3*c,d*). These patterns were consistent between mixed-longitudinal and within-individual analyses and indicated that network narrowing, i.e. the reduction in grooming partners, was not compensated by increased investment in remaining partners, which is incompatible with scenario (*a*) of selectivity (figure 1*a*).

We modelled the frequency at which a female approached each partner in a season-year as a function of her age and cumulated previous DSI with the partner. In the mixed-longitudinal model, the slope for age was negative, the slope for cumulated previous DSI positive, and the interaction of the two not significant (electronic supplementary material, table S1). Therefore, females were selective as they approached those partners more with whom they had shared a stronger tie in the past (figure 3*g*), an effect that was not moderated by age. The model differentiating between within- and between-individual effect corroborated these results; previous relationship strength was a positive predictor of approaching a partner which was not affected by age changes within the individual (electronic supplementary material, table S8). In summary, females exhibited a decreasing frequency of approaches towards partners, even those with whom they had a higher cumulative DSI. Despite this decrease, they maintained a selective inclination towards stronger ties, thereby refuting scenario (*b*) in favour of scenario (*c*). We note that effects of cumulative DSI were quite small and conclude that these time series analyses provided some evidence for relatively constant, but not increasing, selectivity across the lifespan (conveyed with '(N)' in table 1).

These findings indicate that the decrease in both the number of partners groomed and the time spent grooming did not align with a concurrent tendency to focus on a few specific partners. Instead, it appeared that females actively withdrew from social interactions. This hypothesis was further supported by the net reduction in the frequency with which females approached others, irrespective of the partner's identity (table 1 and electronic supplementary material, table S1). Data showed that a female on average 15 years old would have decreased approaches towards others from 1 to 0.56 per hour over 8 years (figure 3*e,f*).

### (d) Comparison of mixed-longitudinal, within-individual and between-individual effects

Most conclusions drawn from mixed-longitudinal analyses were confirmed at the individual level. Yet, mixed-longitudinal effects tended to underestimate age-related changes in social behaviour, and in some cases diverged from within-individual dynamics. Results altogether suggested that individuals with fewer grooming partners and individuals that groomed and approached others less (i.e. less social individuals) were underrepresented at older ages. These inconsistencies are better understood when comparing age-related changes in sociality within individuals and across individuals of different ages. For grooming outdegree, grooming outstrength, and approaching others, both within- and between-individual slopes were significant and negative, but the between-individual effect sizes were much smaller than the within-individual effects (table 1). More surprisingly, within-individual ageing was associated with a decline in the number of partners grooming the subject, in contrast to a tendency to increase across individuals. This suggests that the between-individual effect masked the decrease experienced by individuals with age when the pattern was investigated mixed-longitudinally without differentiating within- and between-individual effects. We note, however, that the significant difference in the slope of within- and between-individual effects on the frequency of being approached by others would suggest the opposite, namely that more social individuals died at an earlier age (although the between-individual effect for this variable was far from significant). Finally, within- and between-individual slopes did not differ for selective approach, instrength and time spent alone, although here again between-individual effects were small compared with within-individual changes.

## 4. Discussion

This study provides evidence for social ageing among females of a wild population of Assamese macaques. By investigating changes in several social behaviours with age, we tested four hypotheses on drivers of social ageing. With advancing age, females did not spend more time alone isolated from others, providing no support for increasing spatial segregation with age. Yet, as females aged, they approached others less frequently, groomed fewer partners, and spent less time grooming others. Females were socially selective by approaching more often those partners they shared a stronger tie with, and this tendency was maintained, but not increasingly expressed, into old age. Finally, females were not less frequently approached or groomed by others, but received grooming from increasingly fewer partners as they aged, consistent with some aspects of social exclusion. Together, these results provide evidence for the contribution of both active and passive processes in the reduction of a female's social network and engagement with advancing age. Furthermore, age effects were weaker between than within individuals, and consequently, age-related changes identified by mixed-longitudinal analyses did not always match true ageing effects. Here, we first discuss what could explain such active and passive social changes, then what these results bring to comparative ageing in light of the social selectivity hypothesis, before ending with a consideration of the implications of studying ageing longitudinally.

Changes experienced with advancing age are not limited to the social domains and most evidence in wildlife shows alterations in body condition and reproductive function [43]. For example, ageing females may modify their activity budget, reducing social time to free time for resting and feeding [9,13,44]. An increase in time spent resting could explain a decrease in time spent actively grooming others and in the number of partners groomed but would not be sufficient to explain a decrease in the number of partners grooming the ageing subject as recipients of grooming are resting. Additionally, changes in the activity budget may be reflected in different spatial distributions [45]. Although ageing was not associated with increasing time spent alone, older females may remain within 5 m proximity to others while engaging in other activities, like feeding, without initiating grooming interactions. Indeed, age was associated with a tendency to approach others within 1.5 m less often, suggesting that females were less motivated to interact with those around them or that they found themselves close to others when the context was not favourable to grooming.

Active social behaviour may also decrease if older individuals experience a decline in the ability to digest food and extract energy due to reduced gut integrity, disruption of gut microbiome communities, or infection with macroparasites (e.g. helminths) [46,47]. Such gastrointestinal disorders may further influence passive social behaviour if group members detect signs of infections (e.g. lethargy or modified faecal odours) and avoid infected conspecifics, as shown in mandrills and Barbary macaques [48,49]. In this population of female Assamese macaques, age is associated with an increased abundance in the gut of several pro-inflammatory bacterial taxa and the oldest females have a lower stability of gut bacterial composition [50], which could impact gut health and contribute to both the active and passive decrease in grooming partners. The linear relationships between age and the social traits observed here do not exclude the possibility of an additional age-independent deterioration among the oldest which could more closely match physiological decline. For example, body mass decreases linearly with age to a point where it drops more rapidly before death in ungulates and yellow-bellied marmots [51,52]. A larger sample size of subjects at old ages is needed to test whether non-human primates experience accelerated social decline in late life.

Social disengagement could also arise from external, rather than internal frailty-related, constraints, if ageing females find themselves in increasingly unfavourable social environments. Non-human primates often show a preference to interact with close kin [13,17,23] or individuals of similar age [13,15]. The availability of such partners will depend on stochastic demographic events like group splits or the death of same-aged individuals, which then constrains the ability to focus on preferred partners. However, current evidence does not suggest that kin availability decreases with age in cercopithecines [53,54], and we did not examine the influence of the death of a close partner because, with the exception of spousal death in humans [55], affiliative networks in humans [56], non-human primates [17,57] and ungulates [7] appear to be resilient to partner loss. In summary, investigations of within-individual changes in non-social behaviour and health appear to be the most fruitful follow-up to pinpoint the causes for active and passive social ageing in this population.

Although frailty and declining motivation may contribute to decreasing social engagement with age, prevailing socioemotional selectivity theories of ageing tend to emphasize increasing selectivity in partner choice as a driver of age-related network narrowing [4–6,56]. According to socioemotional selectivity theories, among humans changes in network composition and size result from greater priority given to maintaining emotionally positive relationships over acquiring new information, leading subjects to discard less valued relationships. Evidence for a greater investment in two-sided rather than one-sided grooming partners [15], reduced interest in non-social compared with social information [14], and an increase in the proportion of kin in the network in non-human primates suggest that preferential interest may shift with age in other species too. How such a shift could account for the reduction in social engagement and network size with age in several species [7,11,14,16] remains unclear, especially because similar social patterns are expected irrespective of whether social selectivity or general decline is assumed (figure 1). We have proposed three scenarios of selectivity, with two (*a* and *b*; figure 1) considering selectivity as a driver of network narrowing and reduction in overall time spent interacting socially. Contributing to answering this question, our findings corroborate the reduction in network size and social engagement with age, and further provide evidence that these changes occur within individuals, emphasizing the social dimension of ageing in wild populations. However, selectivity did not account for this decline, which echoes previous evidence of concomitant decrease in grooming given and preserved selective interest in cues from preferred partners in another primate [14], and contrasts with patterns more closely related to scenario (*a*) [15,43]. In the latter two cases, overall time spent social did not decrease with age. However, this would likely not explain the differences between findings, as the scenarios proposed in figure 1 decouple overall time spent interacting socially from the expression of selectivity. Taken together, these results suggest that selectivity and engagement may be two social dimensions that remain independent into old age at a time when other constraints lead the ageing subject to disengage from most social interactions.

One of the aims of the social selectivity hypothesis of ageing was to suggest a counter-narrative to previous theories considering social disengagement as a detrimental process. Older individuals may not experience detrimental consequences of social disengagement if partners continue investing in them. In Assamese macaques, remaining partners appeared to compensate for the decrease in the number of partners grooming the subject, resulting in mostly constant grooming received. The overall time spent grooming from combined active and passive processes nevertheless decreased with age (electronic supplementary material, figure S7). Although decreasing social engagement with age may be described with loaded terms such as ‘decline’ or ‘disengagement’, the consequences of such disengagement may be beneficial, which has not been conclusively tested yet. There is repeated evidence that the effect of social bonds and integration on physiology and health is modulated by individual dominance status [58], reproductive state [59], sex or dominance status of the social partner [59,60], or by context-dependencies, such as timing of mating season or group-level social stability [61]. Similarly, the social or emotional benefits derived from social interactions could differ across the lifespan, resulting in several age-specific optima. This hypothesis has only rarely been directly addressed, and results are inconclusive [29,62,63]. Describing how social bond strength, social status and broader social integration vary for specific age-classes will be a necessary first step requiring large enough cohorts at different ages. It is further important to formulate hypotheses regarding which social system or environmental conditions should favour increasing, or conversely decreasing, benefits of sociality with age.

Critical to the study of ageing in wild populations, the comparison of mixed-longitudinal and within-individual effects of age revealed that changes within-individual were partially confounded by demographic processes at the population level. For example, the decrease in the number of partners grooming the subjects was only discovered using a longitudinal analysis. The difference between the within-individual (i.e. senescence) and between-individual (i.e. demographic) effects of age on social traits would suggest selective disappearance of females that groomed and were groomed by fewer grooming partners and groomed overall less. In other free-ranging populations selective disappearance was demonstrated for lighter individuals in ungulates [52] and mouse lemurs [19], poorer breeders in great tits [64], and subordinates in meerkats [65], but not in rhesus macaques [17] and wolves [66]. It has to be noted, though, that in the Assamese macaques between-individual effects were generally small, perhaps because sampling was limited across the within-individual age range [42]. The oldest subjects were already mid-aged adults at the beginning of the study, which implies that age at the start and at the end of the study are not independent. A more direct test of selective disappearance is achieved if all individuals enter the study at the same age and are followed until death [67]. More confidence can be placed in the estimates of the within-individual slopes which are robust to model choices [42].

What could explain the disappearance at younger ages of females with fewer grooming partners, approaching others less, but being approached more? Social integration has been positively linked to survival in mammals [3,68], in part mediated by enhanced access to food resources [69], more central positions within groups associated with lower predation-related mortality [8,65,70], resistance to disease [47], and buffering against adverse social and abiotic environments [71–73]. In this population, females mitigate intra-group competition by distancing themselves when feeding, which may come at the expense of an increased predation risk [24]. However, time spent alone only increased slightly across individuals with advancing age, suggesting that those more frequently observed alone were also on average older, inconsistent with increased mortality from predation at the periphery. Females also navigate feeding competition by preferentially attending food patches with females with whom they frequently groom [74], which could lead to sociality-dependent energy intake and survival of those with better body condition [19], whereas frequency of being approached could increase if females are more frequently displaced during those feeding bouts. This is entirely speculative, and we would therefore predict the value of these social bonds to be especially high during gestation and late lactation, when females increase feeding to meet their energetic requirements [26], which could be tested by assessing the relationship between social integration and timing of death in the population.

The possible coexistence of senescence and selective disappearance in shaping age–sociality dynamics is critical for interpreting the results of previous mixed-longitudinal studies. Active engagement in social interactions, measured as either grooming given or a combination of grooming and approaching others, was found to decrease ([8–11,13,14,16], females [12,75]) or remain stable (male chimpanzees [12,15]) in several non-human primates with age. Passive engagement was found to increase (one of two social groups [9], males [75]), decrease ([11,16], females [75], males [12], one of two social groups [9]), or stay stable ([14,15], females [12]). Finally, studies reporting undirected measures of social engagement found a decrease [9,13,71] (or a decreasing trend [76]) or no change [44,70,77] with age. Combining all metrics (and counting sexes from the same study separately), 12 out of 17 studies report at least one negative relationship between social engagement and age. Under the (extreme) assumption of no social senescence, the negative correlation between age and social engagement in four wild and eight captive studies would be caused by the selective disappearance of highly socially engaged individuals. Alternatively, assuming some degree of senescence, the absence of a relationship between age and sociality in three wild and two captive populations could have arisen from the selective disappearance of poorly connected individuals. Although speculative, it seems more plausible that unaccounted selective disappearance contributes to discrepancies between studies by masking social senescence.

In conclusion, this study highlights that social ageing occurs in wild primates and, in conjunction with demographic effects, contributes to age-related variation in social behaviour. As females aged, they engaged less frequently in social interactions, which led to a narrowing of their social network despite evidence for selectivity in partner choice. Possibly because of such disengagement, fewer partners interacted with females as they grew older. This effect was partially confounded by demographic trends, compatible with selective disappearance, further highlighting the need to account for demographic effects, a critical point in both animal and human biogerontology research [18,56,64]. Complete life-course trajectories are almost impossible to collect in humans, and mixed-longitudinal studies are biased by selective mortality and sensitive to self-perception and memory recollection biases when surveys are used [4,18]. In this context, longitudinal data collected through systematic direct observation of animal populations represent a valuable contribution to social ageing research.

## Data Availability

Data and code for this manuscript have been deposited to Dryad: https://doi.org/10.5061/dryad.r4xgxd2mq [40]. Supplementary material is available online [78].
